# Molecular mechanisms underlying the evolution of the slp76 signalosome

**DOI:** 10.1038/s41598-017-01660-0

**Published:** 2017-05-04

**Authors:** Xuemei Qu, Xin Lan, Chong Deng, Jiatao Zhou, Jingjing Du, Shengfeng Huang, Yingqiu Li

**Affiliations:** State Key Laboratory of Biocontrol, Key Laboratory of Gene Engineering of the Ministry of Education, College of Life Sciences, Sun Yat-Sen University, Guangzhou, 510275 People’s Republic of China

## Abstract

The well-defined mammalian slp76-signalosome is crucial for T-cell immune response, yet whether slp76-signalosome exists in invertebrates and how it evolved remain unknown. Here we investigated slp76-signalosome from an evolutionary perspective in amphioxus Branchiostoma belcheri (bb). We proved slp76-signalosome components bbslp76, bbGADS and bbItk are present in amphioxus and bbslp76 interacts with bbGADS and bbItk, but differences exist between the interaction manners within slp76-signalosome components of amphioxus and human (h). Specifically, bbslp76 has a unique WW-domain that blocked its association with hItk and decreased TCR-induced tyrosine-phosphorylation and NFAT-activation. Deletion of WW-domain shifted the constitutive association between bbslp76 and hPLCγ1 to a TCR-enhanced association. Among slp76-signalosome, the interaction between slp76 and PLCγ1 is the most conserved and the binding between Itk and slp76 evolved from constitutive to stimulation-regulated. Sequence alignment and 3D structural analysis of slp76-signalosome molecules from keystone species indicated slp76 evolved into a more unfolded and flexible adaptor due to lack of WW-domain and several low-complexity-regions (LCRs) while GADS turned into a larger protein by a LCR gain, thus preparing more space for nucleating the coevolving slp76-signalosome. Altogether, through deletion of WW-domain and manipulation of LCRs, slp76-signalosome evolves from a rigid and stimulation-insensitive to a more flexible and stimulation-responding complex.

## Introduction

After TCR ligation by the peptide-MHC complex on APC, the lymphocyte specific protein tyrosine kinase (Lck) is activated and phosphorylates the immunoreceptor tyrosine-based activation motifs (ITAMs) of CD3 complex subunits, thereby facilitating the recruitment and activation of the CD3ζ chain-associated protein of 70 kDa (Zap70) kinase. The Recruitment of Zap70 leads to a cascade of phosphorylation events involving linker for activation of T cells (LAT), SH2 domain-containing leukocyte protein of 76 kDa (slp76), protein kinase C-θ (PKCθ) and other signaling molecules, resulting in the activation of a number of transcription factors, notably NFAT, NF-κB and AP-1, and subsequent interleukin 2 (IL-2) production and T cell proliferation (reviewed in refs 1–3). The slp76 adaptor nucleates a large signaling complex (slp76 signalosome), which is mainly comprised of slp76, Grb2-related adaptor downstream of Shc (GADS), interleukin- 2-inducible T cell tyrosine kinase (Itk), phospholipase C-γ1 (PLCγ1), VAV1 and NCK^4–7^. While Itk directly activates PLCγ1^8, 9^, the adaptor slp76 regulates PLCγ1 activation through manipulating effector protein interactions and localizations. Deficiency in almost any one of the slp76 signalosome components disrupts PLCγ1 activation, leading to the defect in calcium mobilization and NFAT activation. slp76 contains a sterile α motif (SAM) domain, a central proline rich region (PRR), a carboxy-terminal SH2 domain and four tyrosine phosphorylation motifs^5, 10^. Upon TCR stimulation, the three N-terminal tyrosines of slp76, Y112, Y128 and Y145 are phosphorylated by Zap70^11, 12^. Through its PRR, slp76 binds to the SH3 domain of LAT-associated GADS, which illustrates how slp76 is recruited to LAT^13–17^. The association between slp76 Y145 and Itk-SH2 brings Itk into close proximity to LAT-bound PLCγ1^18–20^. slp76 also interacts with the SH3 and C-terminal SH2 of PLCγ1 by its PRR and Y173, an Itk-targeted tyrosine of which phosphorylation depends on the three N-terminal tyrosines and primes PLCγ1 for activation^19, 21, 22^. All these intermolecular interactions among the slp76 complex are indispensable for proper TCR signaling closely related to T cell development and activation. Blocking GADS-slp76 interaction disrupted slp76 localization and T cell function^15^. A continued binding of Itk to slp76 is required to keep Itk in an active state^23^. Although this TCR-induced slp76 signalosome is well characterized in mammalians, whether it is evolutionarily conserved in lower organisms and how the molecules within the complex evolved to better adjust to one another, preparing higher organisms for finer signaling regulations, are unknown.

Amphioxus, a chordate invertebrate linking nonchordate lineage and vertebrate lineage, serves as one of the best models for understanding the vertebrate ancestral immunity. Although there is no evidence of the presence of V(D)J recombination in amphioxus so far, the homologs of Recombination activation gene 1 (RAG1) core domain and its N-terminal domain, RAG2 as well as the RAG1 gene activator have been found in amphioxus genome. It is also demonstrated that amphioxus has lymphocyte-like cells and primitive adaptive-immunological molecules (reviewed in ref. 24). Recently, the structure of a Variable Lymphocyte Receptors (VLR) like receptor protein was identified in amphioxus^25^. However, the homologs of TCR-proximal molecules such as Zap70, Lck and slp76 have not been reported in amphioxus, to our knowledge.

Here, we cloned bbslp76, bbGADS and bbItk and investigated their intermolecular interactions as well as those with their human counterparts. We found that the slp76/GADS/Itk complex is conserved in amphioxus. However, neither bbslp76 nor bbGADS performs properly in human T cells, suggesting a distinct interaction pattern within bbslp76 signalosome. We also determined that the association between slp76 and PLCγ1 is the most evolutionarily conserved and the constitutive Itk-slp76 binding evolved to stimulation-induced interaction. Given the sequential and 3D structural analysis, we propose a model for the evolution of the slp76 complex that, as scaffolds, slp76 discards its dispensable domain evolving into a more unfolded structure and GADS develops into a larger protein in the course of evolution, all of which expands the space between LAT and slp76 to prepare vertebrate Itk and PLCγ1 for better recruitment to the LAT/slp76 signalosome.

## Results

### Sequence and phylogeny tree of bbslp76

**Figure 1:**
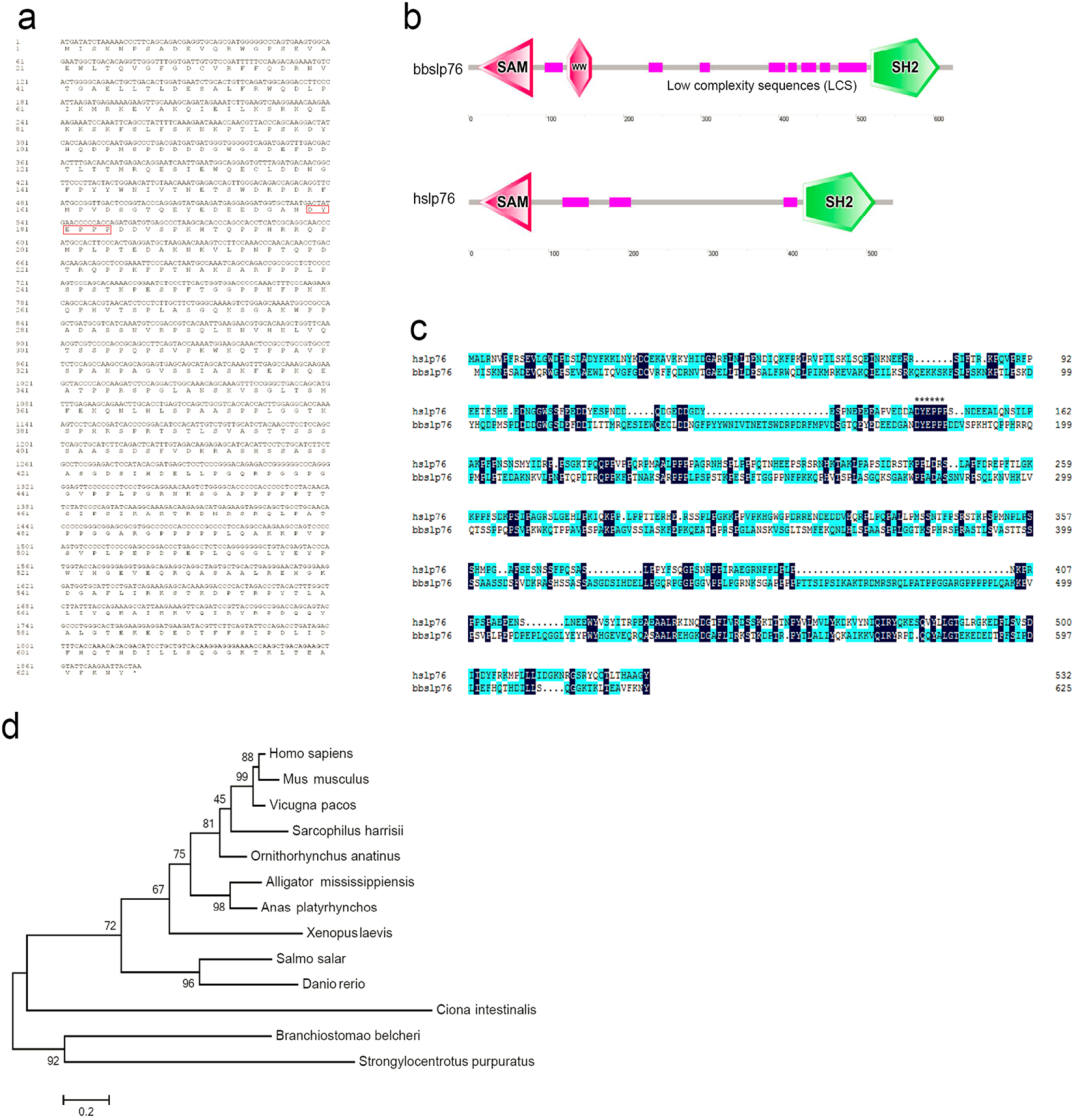
Sequence and phylogeny tree of bbslp76. (**a**) Nucleotide and amino acid sequence of bbslp76. Itk binding motif DYEPPP was marked in the red frame. (**b**) Protein structures of bbslp76 and hslp76 generated by SMART. (**c**) Sequences alignment of bbslp76 and hslp76. The color of Sky blue represents 50% consensus of amino acid and dark blue represents 100% consensus of amino acid. Itk binding motif DYEPPP motif was highlight with ******. (**d**) Phylogenetic tree of slp76. The neighbor-joining phylogenetic tree was constructed on the basis of 13 different slp76 sequences from GenBank, utilizing the sequence analysis tool MEGA 5.

A full-length cDNA of 1926 bp was obtained from a *Branchiostoma belcheri* cDNA library (Fig. 1a), encoding a polypeptide of 642 amino acids with an N-terminal SAM domain, a WW domain adjacent to the SAM domain, a C-terminal SH2 domain, and a central PRR (Fig. 1b). This protein shares the highly conserved SAM and SH2 domains with hslp76 and was designated as *Branchiostoma belcheri* slp76 (bbslp76) (Fig. 1b). Compared with hslp76, the WW domain in bbslp76 was revealed to be a unique domain (Fig. 1b). The overall sequence identity of bbslp76 with hslp76 is 33% and the N-terminal Zap70-targeted Y145 of hslp76, which is also known as the hItk binding motif (DYEPPP)^26^, is conserved in bbslp76 (Fig. 1c). The phylogenetic tree showed that the evolutionary position of bbslp76 shares a common ancestor with vertebrate slp76, which is in accordance with the transition status of amphioxus in the evolution of chordates (Fig. 1d). Thus, the characterization of bbslp76 would help to study the evolution of slp76 and the adaptive immunity.

### Tissue-specific expression patterns of bbslp76

**Figure 2:**
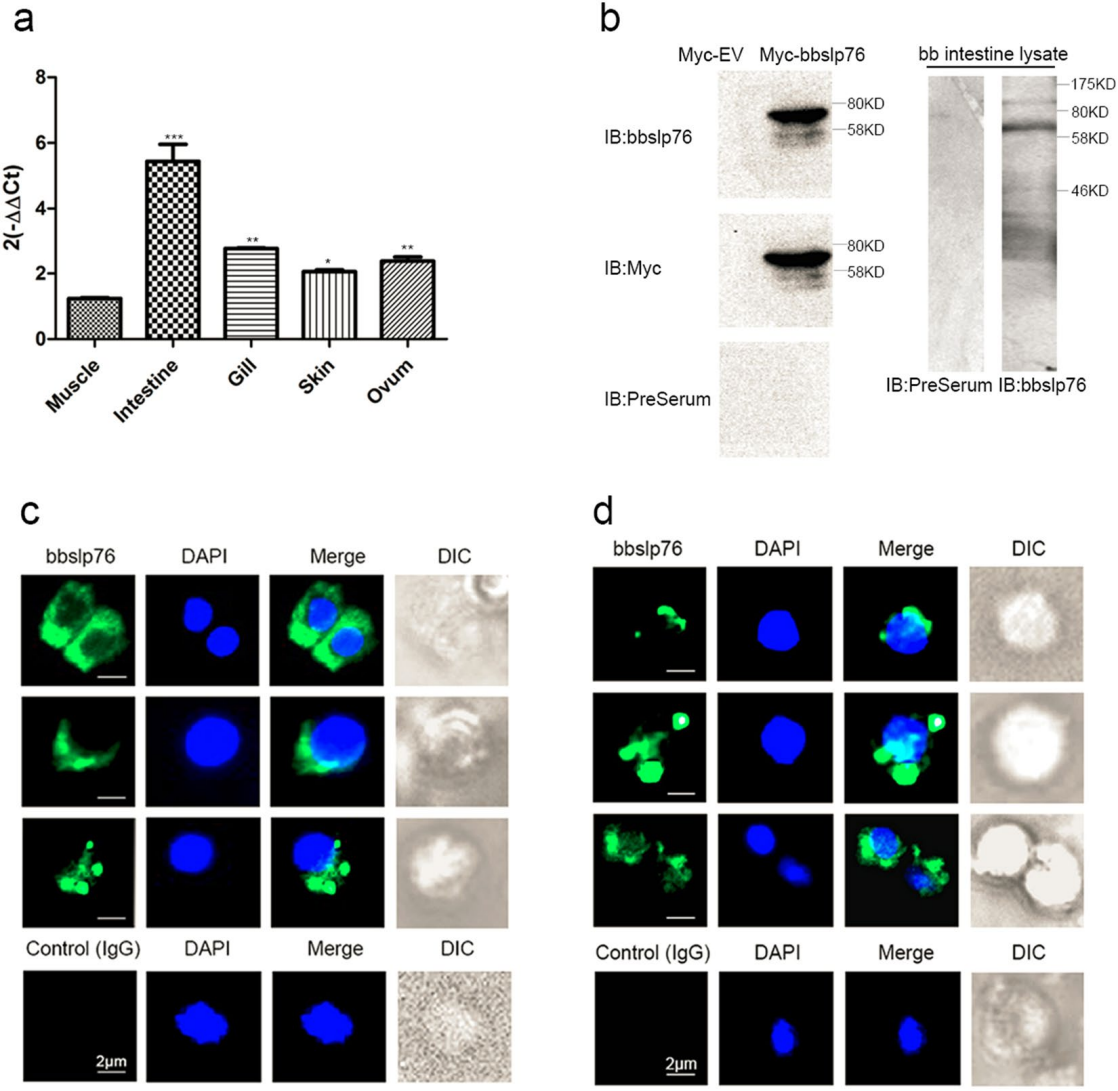
Tissue expression patterns of bbslp76. (**a**) Relative expression levels of bbslp76 gene in various tissues of *B. belcheri* by qRT-PCR. 2-ΔΔCt values (normalized to endogenous control 18S) represented the expression of the bbslp76 gene in various tissues (muscle, intestine, gill, skin, ovum) of *B. belcheri*. Bars represent the average results of triplicate reactions for each tissue. Error bars represent the standard error of the mean (SEM) among three biological replicates. *, ** and *** indicate a significant expression level difference versus muscle as control at p < 0.05, p < 0.005 and p < 0.005, respectively. (**b**) bbslp76 polyclonal antibody titration (1:1000) in 293 T cells with exogenous expression of cMyc-bbslp76 and in amphioxus intestine lysate respectively. EV: Empty Vector﻿. (**c**) and (**d**) Localization of bbslp76 in the gill (**c**) or intestine cells (**d**) of amphioxus. Amphioxus gill or intestine cells were bound to poly-L-Lysine-coated coverslips, fixed, and stained with anti-bbslp76 polyclonal antibody followed by a secondary fluorescent antibody (green). Nuclei were stained with DAPI (blue). Images were acquired using fluorescent microscopy at 400 times magnification. Bar = 2 μm.

As determined by qRT-PCR, bbslp76 mRNA levels varied across different tissues including muscle, intestine, gill, skin and ovum in individuals. The intestine had the highest mRNA level of bbslp76, followed by gill (Fig. 2a). This mRNA expression profile of bbslp76 is consistent with the previous finding that intestine and gill are two potential amphioxus immune organs^27, 28^. By immunizing mice with the bbslp76 (1-318aa) polypeptide, we prepared the polyclonal antibody of bbslp76 that could specifically detect both the exogenous (Fig. 2b left) and endogenous (Fig. 2b right) bbslp76 with a band of approximate 70KD in contrast to the Myc-EV and preimmune serum, respectively. The molecular weight of bbslp76 was further determined by the Myc antibody (Fig. 2b left). Next, we used the homemade polyclonal antibody of bbslp76 for the immunofluorescent staining of amphioxus intestine cells and gill cells. The results further demonstrated bbslp76’s expression in the two tissues and showed that bbslp76 was mainly clustered in the cytosol, although there were cases where bbslp76 was evenly distributed (Fig. 2c,d). And the cells where bbslp76 formed clusters (Fig. 2c, the 2^nd^, 3^rd^ panel, and Fig. 2d, the 1^st^, 2^nd^ panel) were bigger than those with bbslp76 evenly distributed (Fig. 2c, the 1^st^ panel and Fig. 2d, the 3^rd^ panel). It’s reported that after the microbial challenge, the lymphocyte-like cells in amphioxus gills are bigger than those of normal cells^29^. Additionally, after TCR ligation, signaling components at the cell-cell contact surfaces including hslp76 assemble into microclusters which serve as primary sites for early tyrosine phosphorylation events^30, 31^. Therefore, we propose that bbslp76 may cluster in activated lymphocyte-like cells while present an even distribution in resting ones. These findings suggest that bbslp76 may be involved in amphioxus immune regulation.

### bbslp76 inhibits TCR-induced NFAT activation

**Figure 3:**
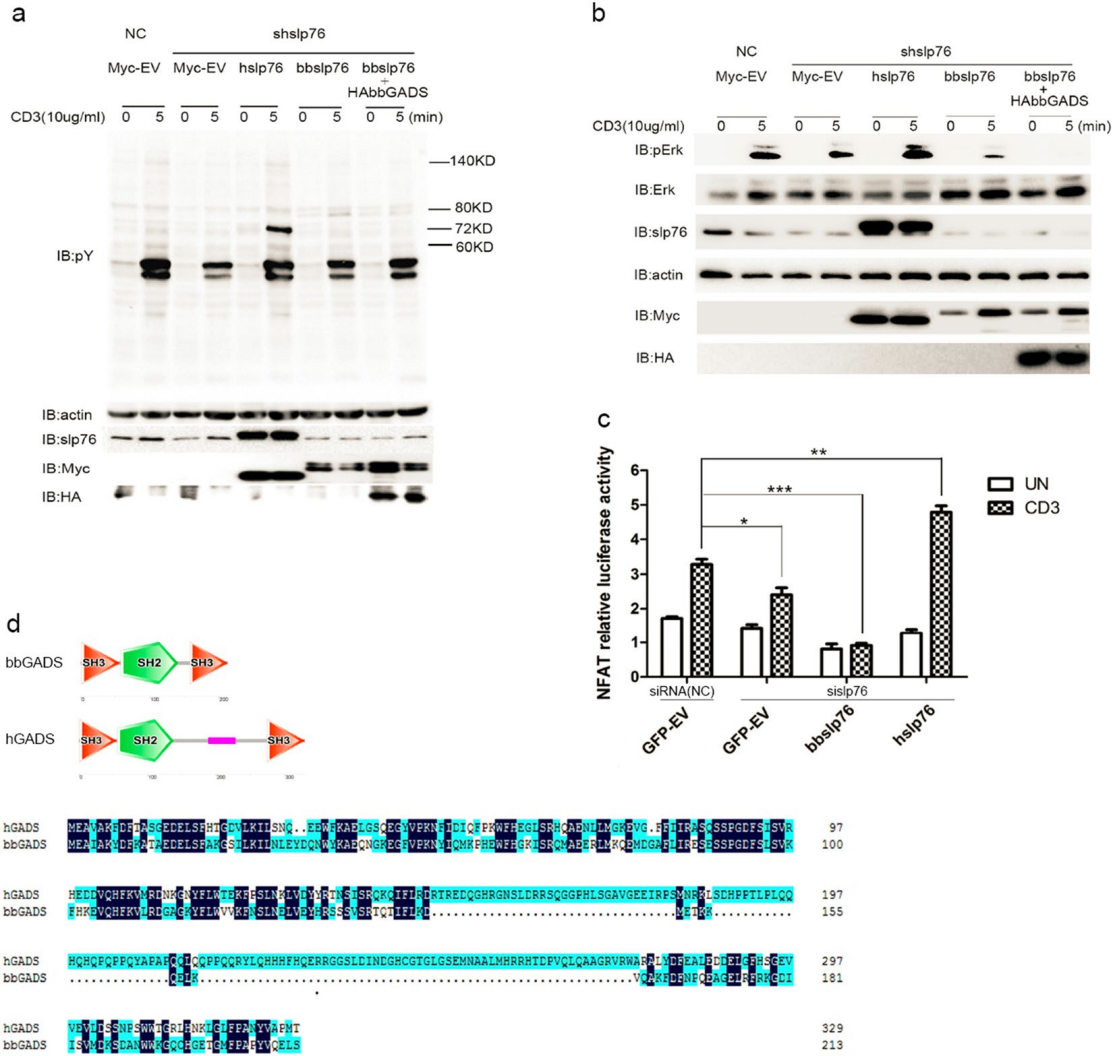
bbslp76 inhibits TCR-induced NFAT activation. (**a**) bbslp76 inhibits the total tyrosine phosphorylation of Jurkat TAg cells after TCR ligation. Jurkat TAg cells were transfected with NC (negative control, scrambled small interfering RNA) and shslp76 (short hairpin RNA targeting hslp76 RNA specifically). shslp76-transfected cells were separately co-transfected with Myc-hslp76, Myc-bbslp76, and Myc-bbslp76 plus HA-bbGADS. After 48 hrs, cells stimulated with or without CD3 (10 μg/mL, 5 min) were subjected to western-blotting to analyze the tyrosine phosphorylation (pY) of T cells. (**b**) bbslp76 inhibits the phosphorylation of Erk (pErk) in Jurkat TAg cells after TCR ligation. Cells and lysates were prepared as in (**a**). (**c**) bbslp76 inhibits NFAT activity in T cells. Jurkat TAg cells were transfected with NC and sislp76 (small interfering RNA targeting hslp76 RNA specifically). NC and sislp76 cells were transfected with an NFAT reporter construct and a co-transfected Renilla luciferase and the indicated constructs. 48 hrs later, cells were untreated or stimulated with CD3 (3 μg/mL) for 8 hrs and lysed for the reporter gene assay. Luciferase activity was normalized using a co-transfected Renilla luciferase. The data represent the mean ± SD from triplicate determinations. *P < 0.05, **P < 0.005, ***P < 0.0005. (**d**) Protein structure of bbGADS and hGADS generated by SMART. And sequences alignment of bbGADS and hGADS. The color of Sky blue represents 50% consensus of amino acid and dark blue represents 100% consensus of amino acid. The experiments were repeated three times with very similar results.

To explore the function of bbslp76 in TCR signaling, we transiently transfected Jurkat TAg cells with hslp76 or bbslp76 together with shslp76 (a specific small hairpin RNA targeting the 3′ untranslated region of hslp76 mRNA) expression vector. As shown in Fig. 3a,b, the overall TCR-induced tyrosine-phosphorylation (pY) of TCR-proximal signaling proteins and the phosphorylation of downstream Erk (pErk) were decreased in T cells by shslp76. Co-expression of hslp76 but not bbslp76 blocked this shslp76-induced decline in overall pY and pErk (Fig. 3a,b). As hslp76 is an essential regulator of TCR-induced NFAT activation, we further tested the effect of bbslp76 on NFAT activation. When hslp76 was knocked down with sislp76 (a specific small interfering RNA targeting the 3′ untranslated region of hslp76 mRNA) in Jurkat TAg cells, the NFAT activity was severely dampened compared with that in T cells transfected with a scrambled siRNA as negative control (NC), and the exogenous expression of hslp76 but not bbslp76 restored the sislp76-induced deficiency in NFAT activation (Fig. 3c). hGADS is required for hslp76’s association with hLAT and recruitment of hPLCγ1^32^. The inhibition on TCR signaling caused by bbslp76 may be resulted from the dysfunction of amphioxus GADS (bbGADS) which further leads to bbslp76’s deficiency in interacting with hLAT and hPLCγ1 in Jurkat TAg cells. To investigate whether bbGADS can rescue the activation of TCR signaling, we cloned bbGADS which encodes a polypeptide of 213 amino acids with a highly conserved protein structure comprising an N-terminal SH3, a C-terminal SH3 domain, and a central SH2 domain (Fig. 3d). Although bbGADS and hGADS have similar structures, hGADS has a LCR between its SH2 domain and its C-terminal SH3 domain, which is absent in bbGADS (Fig. 3d). The overall sequence identity between bbGADS and hGADS is 42% (Fig. 3d). Next, we co-transfected Jurkat TAg cells with bbslp76 and bbGADS and found that bbGADs could not reverse the bbslp76-induced inhibition of the overall pY or pErk (Fig. 3a,b). These results indicated that bbslp76 could not substitute for hslp76 in TCR-induced T cell activation and the slp76 signalosome members may have co-evolved to better adjust to the evolving cellular function.

### The interactions between bbslp76 and TCR-proximal signaling proteins

**Figure 4:**
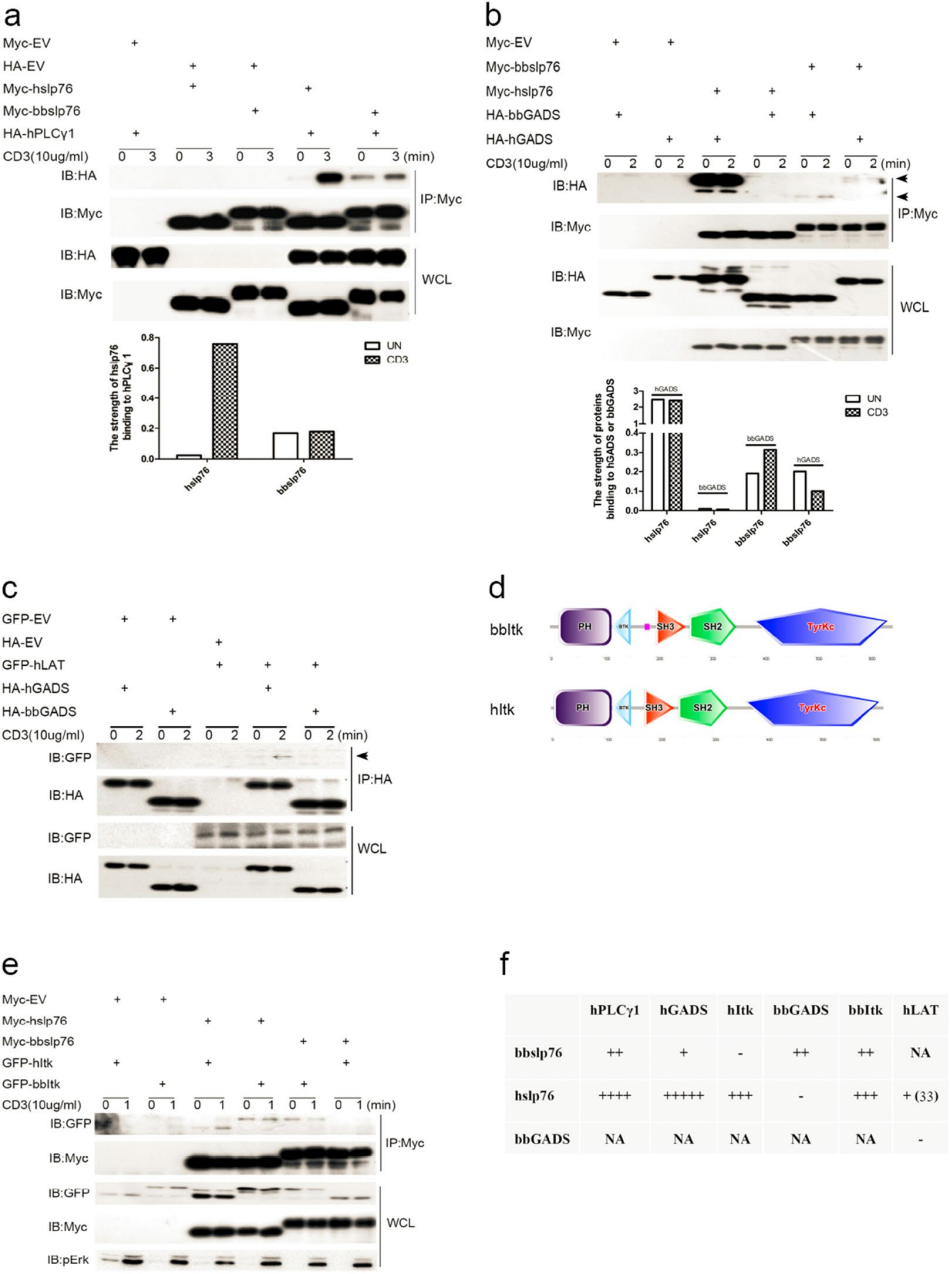
bbslp76 interacts with TCR proximal signal proteins. (**a**) bbslp76 interacts with hPLCγ1 in T cells. Jurkat TAg cells were transfected with the indicated constructs. After 48 hrs, cells were stimulated with or without CD3 (10 μg/mL, 3 min); then, lysed and lysates were split into two parts, one part of the lysates was subjected to a Myc IP (immunoprecipitation), and the other part was used for WCL (whole cell lysis, 40 μL). Samples were studied by western blot, and probed with indicated antibodies. The binding affinity is normalized by IB HA to IP Myc. (**b**) The interaction of bbslp76 or hslp76 with bbGADS or hGADS in T cells. Cells and lysates were prepared as in (**a**). Samples were studied by western blot, and probed with indicated antibodies. The upper arrow indicates hGADS. The lower arrow indicates bbGADS. The binding affinity is normalized by IB HA to IP Myc. (**c**) bbGADS does not interact with hLAT in T cells. Cells and lysates were prepared as in (**a**). One part of the lysates was subjected to a HA IP. Samples were studied by western blot, and probed with indicated antibodies. The arrow indicates hLAT. (**d**) Protein structures of bbItk and hItk generated by SMART. (**e**) bbslp76 does not interact with hItk in T cells. Cells and Lysates were prepared as in (**a**). Samples were studied by western blot, and probed with indicated antibodies. (**f**) Summary of binding affinities between bbslp76 and TCR proximal signal proteins. NA: not available; The experiments were repeated three times with very similar results. The strength of protein-protein interaction was analyzed by software Quantity One.

To understand the differences between the slp76 signalosomes of amphioxus and human, we investigated the interactions among bbslp76 signalosome proteins and their interactions with their counterparts in the hslp76 signalosome. First, the interactions between slp76 and PLCγ1 were studied. In Jurkat TAg cells, both hslp76 and bbslp76 associated with hPLCγ1, however, the binding affinity of hslp76 to hPLCγ1 was much stronger than that of bbslp76 to hPLCγ1. Upon anti-CD3 stimulation, the association between hslp76 and hPLCγ1 was increased. However, this enhanced interaction was not observed between bbslp76 and hPLCγ1 (Fig. 4a), suggesting that the evolved hslp76 enables the evolution from the constitutive binding between slp76 and PLCγ1 to stimulation-regulated binding. Next, we explored the associations between slp76 and GADS. As shown in Fig. 4b, bbslp76 interacted with both bbGADS and hGADS but the binding between bbslp76 and bbGADS or hGADS was much weaker than that between hslp76 and hGADS. bbGADS bound to bbslp76 but not hslp76 (Supplemental data Fig. 1a and Fig. 4b). In addition, bbGADS did not associate with hLAT (Fig. 4c). These results suggested that the association between hslp76 and hPLCγ1 or hGADS is conserved, in line with the central role of hslp76 in hPLCγ1 activation. Furthermore, we cloned bbItk and investigated the interactions between slp76 and Itk. bbItk possesses a similar structure with hItk (Fig. 4d). And as shown in Fig. 4e, hslp76 bound to hItk and the binding was enhanced after stimulation. Interestingly, the association between hslp76 and bbItk was stronger than that between hslp76 and hItk in resting Jurkat TAg cells and was not enhanced upon stimulation (Fig. 4e). The same case is with the interaction between bbslp76 and bbItk (Supplemental data Fig. 1b and Fig. 4e), suggesting that the evolved hItk enables the evolution of the constitutive binding between Itk and slp76 to stimulation-regulated binding. Moreover, bbslp76 interacted with bbItk but not hItk, although bbslp76 contains the hItk binding motif (Fig. 4e). The binding results above were summarized in Fig. 4f, and hslp76’s association with hLAT has been reported^33^. In conclusion, bbslp76 interacts with bbGADS and bbItk, and the hslp76-hPLCγ1 module is the most conserved among the hslp76 signalosome in evolution. Additionally, the inhibition of TCR-induced signaling and NFAT activation by bbslp76 could be resulted from the deficient interaction between bbslp76 and hItk.

### Mapping bbslp76 inhibition domain

**Figure 5:**
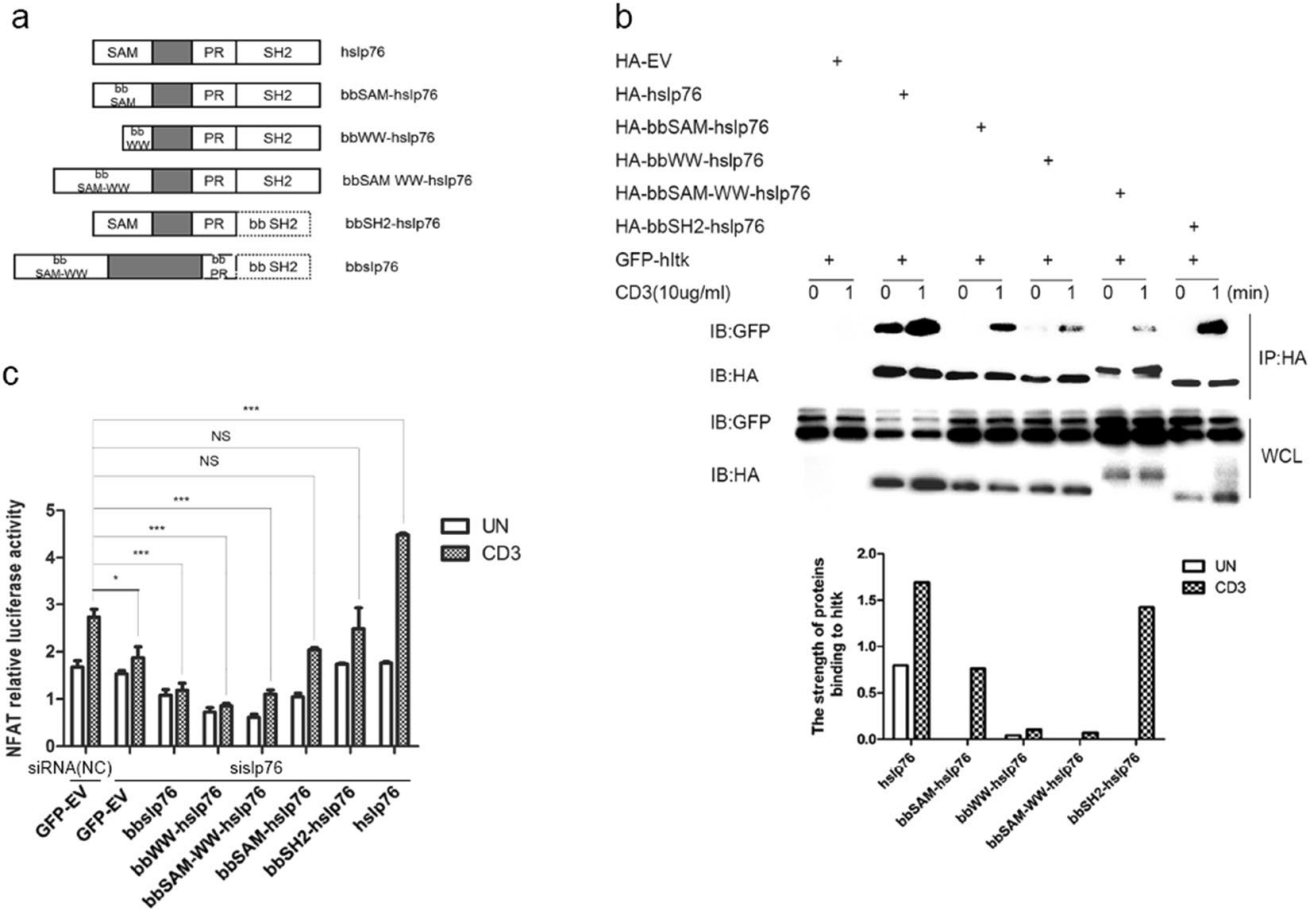
Mapping bbslp76 inhibition domain. (**a**) Schematic structures of chimeric proteins. (**b**) Interactions of chimeric proteins with hItk. Jurkat TAg cells were transfected with the indicated constructs. After 48 hrs, cells were stimulated with or without CD3 (10 μg/mL, 1 min) and then lysed; lysates were split into two parts. One part of the lysates was subjected to a HA IP, and the other part was used for WCL (40 μL). Samples were studied by western blot, and probed with indicated antibodies. The binding affinity is normalized by IB GFP to IP HA. (**c**) bbslp76 inhibits NFAT activity in T cells. Jurkat TAg cells were transfected with NC and sislp76. NC and sislp76 cells were transfected with an NFAT reporter construct and a co-transfected Renilla luciferase and the indicated constructs. 48 hrs later, cells were untreated or stimulated with CD3 (3 μg/mL) for 8 hrs and lysed for reporter gene assay. Luciferase activity was normalized using a co-transfected Renilla luciferase. The data represent the mean ± SD from triplicate determinations. *P < 0.05, **P < 0.005, ***P < 0.0005. The experiments were repeated three times with very similar results. The strength of protein-protein interaction was analyzed by software Quantity One.

To map the sequence on bbslp76 interfering with its association with hItk, we constructed four chimeric proteins (Fig. 5a), including bbSAM-hslp76 (replacing the hslp76 SAM domain with the bbslp76 SAM domain), bbWW-hslp76 (replacing the hslp76 SAM domain with the bbslp76 WW domain), bbSAM-WW-hslp76 (replacing the hslp76 SAM domain with the bbslp76 SAM-WW domain) and bbSH2-hslp76 (replacing the hslp76 SH2 domain with the bbslp76 SH2 domain). Then, we tested the associations between the chimeric proteins and hItk. Our data indicated that the bindings of bbWW-containing proteins to hItk were significantly weaker than those of hslp76 and the other chimeric proteins (Fig. 5b). Additionally, we transfected these constructs into Jurkat TAg cells with hslp76 silenced by sislp76 to investigate the function of bbWW domain in NFAT activation. Compared to NC GFP-EV group with a scrambled siRNA and GFP-EV co-transfected, the knockdown of endogenous hslp76 in the GFP-EV group impaired the NFAT activity, the deficiency of which could not be rescued by bbslp76 or bbWW-hslp76 or bbSAM-WW-hslp76 (Fig. 5c). However, bbSAM-hslp76 and bbSH2-hslp76 successfully restored the decreased NFAT activity, albeit to a less extent than hslp76 did (Fig. 5c). In conclusion, bbWW domain may contribute to the impaired interaction between bbslp76 and hItk and the TCR-induced NFAT activation.

### ΔWW-bbslp76 rescues total tyrosine phosphorylation but partially rescues NFAT activation after TCR ligation in T cells

**Figure 6:**
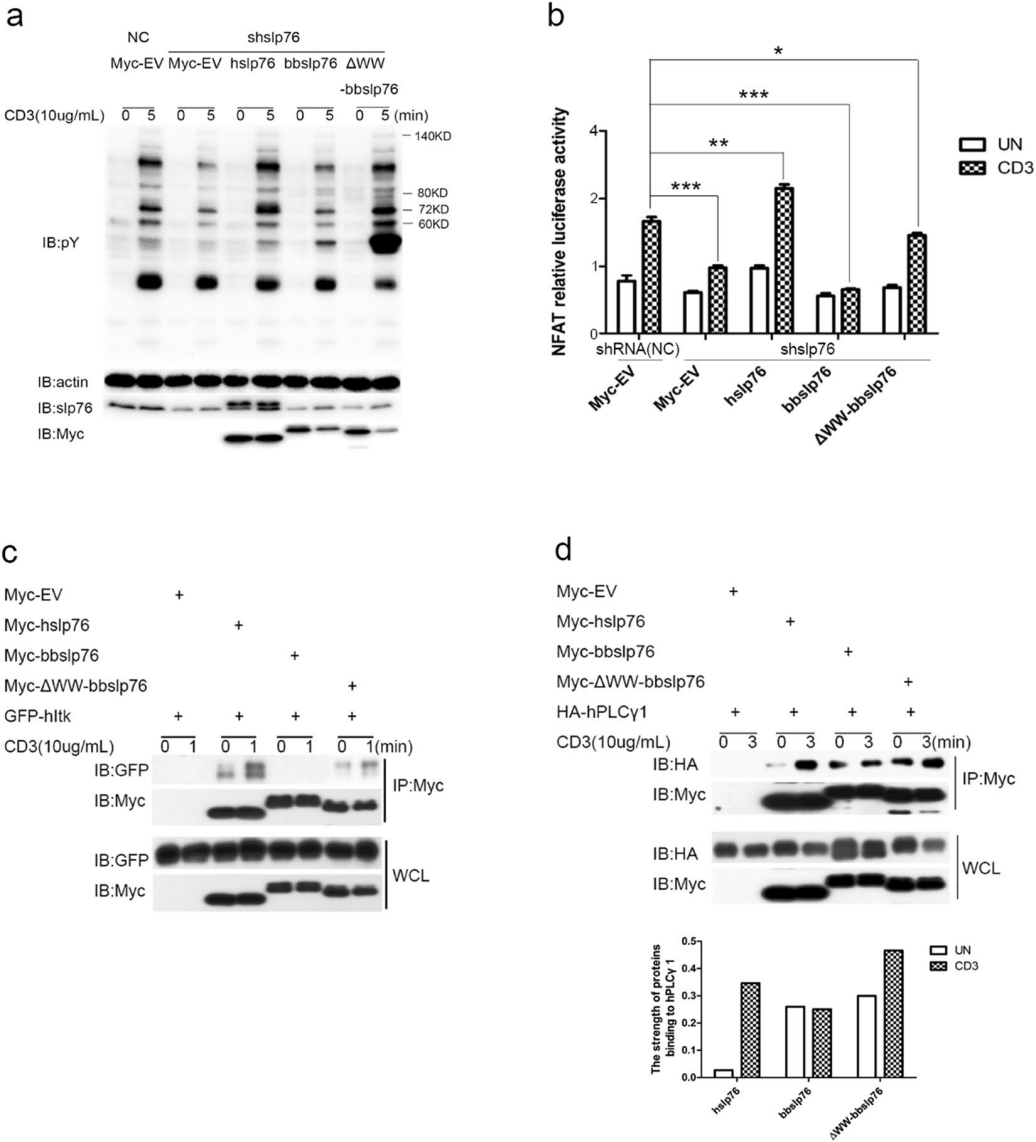
ΔWW-bbslp76 rescues total tyrosine phosphorylation but partially rescues NFAT activation after TCR ligation in T cells. (**a**) ΔWW-bbslp76 rescued total tyrosine phosphorylation in T cells. Jurkat TAg cells were transfected with the indicated constructs and cells and lysates were prepared as in Fig. 3(a). (**b**) bbslp76 partially rescued NFAT activity in T cells. Jurkat TAg cells were transfected with NC and shslp76. NC and shslp76 cells were transfected with an NFAT reporter construct and a co-transfected Renilla luciferase and the indicated constructs. 48 hrs later, cells were untreated or stimulated with CD3 (3 μg/mL) for 8 hrs and lysed for reporter gene assay. Luciferase activity was normalized using a co-transfected Renilla luciferase. The data represent the mean ± SD from triplicate determinations. *P < 0.05, **P < 0.005, ***P < 0.0005. (**c**) ΔWW-bbslp76 interacts with hItk. Jurkat TAg cells were transfected with the indicated constructs. After 48 hrs, cells were stimulated with or without CD3 (10 μg/mL, 3 min) and then lysed; lysates were split into two parts, one part of the lysates was subjected to a Myc IP, and the other part was used for WCL (40 μL). Samples were studied by western blot, and probed with indicated antibodies. (**d**) ΔWW-bbslp76 interacts with hPLCγ1. Jurkat TAg cells were transfected with the indicated constructs. After 48 hrs, cells were stimulated with or without CD3 (10 μg/mL, 1 min). Cells and lysates were prepared as in (**c**). Samples were studied by western blot, and probed with the indicated antibodies. The binding affinity is normalized by IB HA to IP Myc. The experiments were repeated three times with very similar results. The strength of protein-protein interaction was analyzed by software Quantity One.

Since the chimeric proteins containing bbWW domain inhibited their associations with hItk and TCR-induced NFAT activation, we next constructed ΔWW-bbslp76 with the deletion of bbWW domain to test whether this mutant could reverse the inhibition. Jurkat TAg cells were transiently transfected with hslp76, bbslp76 or ΔWW-bbslp76 together with shslp76. The TCR-induced total pY was decreased by shslp76 compared with NC, which could be rescued by the co-expression of hslp76 but not by bbslp76 (Fig. 6a). Interestingly, this decrease could be reversed by ΔWW-bbslp76 as well as hslp76 (Fig. 6a). Consistently, the TCR-induced NFAT activity was severely impaired by shslp76, which was restored by the co-expression of hslp76 but not by bbslp76, and partially rescued by ΔWW-bbslp76 in Jurkat TAg cells (Fig. 6b). Furthermore, our co-immunoprecipitation data showed ΔWW-bbslp76 recovered its interaction with hItk (Fig. 6c). Upon anti-CD3 stimulation, the TCR-induced association of ΔWW-bbslp76 with hPLCγ1 was much stronger than that of bbslp76 with hPLCγ1 and comparable with that of hslp76 with hPLCγ1, yet in resting T cells the binding of ΔWW-bbslp76 with hPLCγ1 was stronger than that of hslp76 with hPLCγ1 (Fig. 6d). Unlike the association between hslp76 and hPLCγ1, the interaction between bbslp76 and hPLCγ1 was not enhanced after stimulation, while ΔWW-bbslp76 rescued the stimulation-responding binding of bbslp76 to hPLCγ1 (Fig. 6d). These results indicated that bbWW domain was responsible for bbslp76’s inhibition of the TCR-induced overall pY and NFAT activation through blocking the association between bbslp76 and hItk.

### 3D model comparison between hslp76, bbSAM-WW-hslp76, bbslp76 and ΔWW-bbslp76

**Figure 7:**
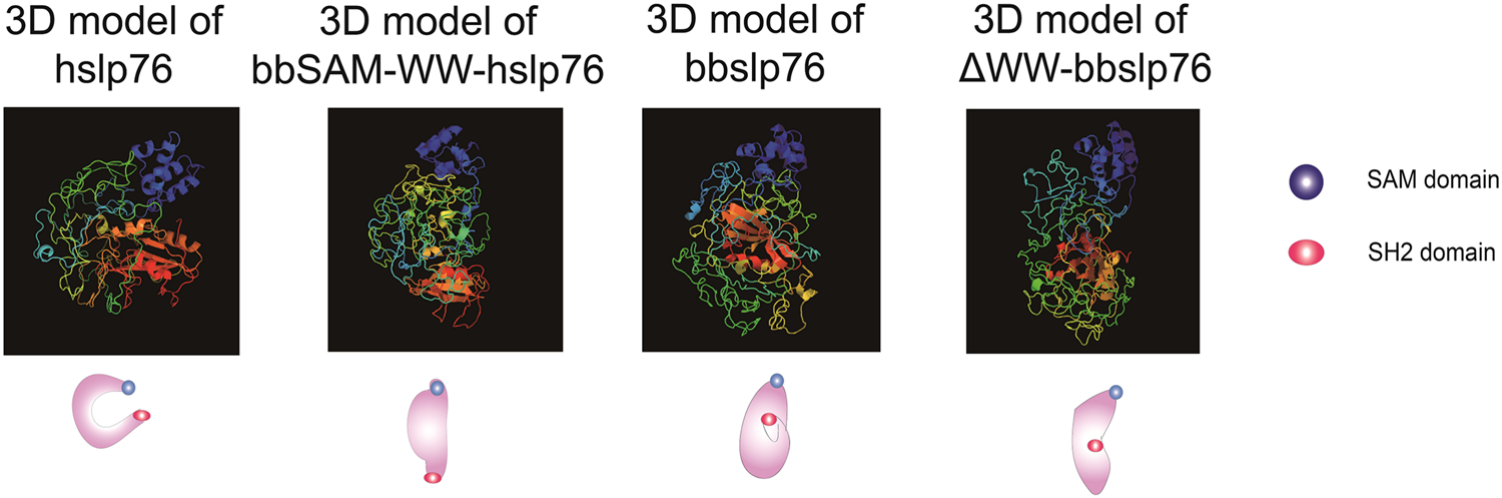
3D model comparison of hslp76, bbSAM-WW-hslp76, bbslp76 and ΔWW-bbslp76. The 3D structures were predicted by Phyre2.

In hslp76, tyrosine 145 lies within a DYEPPP motif and is responsible for hslp76’s binding to hItk. bbslp76 also has the DYEPPP motif but it could not interact with hItk, as we know from the results above. We speculated that bbWW domain may influence the 3D structure of bbslp76 which in turn impairs its association with hItk. To explore the difference of bbslp76 3D structures with or without WW domain, we used Phrey2 online software to predict the 3D model of the wild-type and mutant slp76. The results showed that the hslp76 presents an open and loose C-type-structure with larger contact surface area formed by the sequence between the SAM and SH2 domain (intermediate sequences), which brings much flexibility and elasticity to protein-protein interaction (Fig. 7, the 1^st^ column). The main protein binding sites are fully exposed and decentralized in this structure (Supplemental data Fig. 2). When we substituted the bbSAM-WW domain for the hSAM domain in hslp76, bbSAM-WW-hslp76 forms a closed and compacted structure compared with hslp76 (Fig. 7, the 2^nd^ column). Within this structure, the hPLCγ1 and hGADS binding sites are close and bbWW domain alters the structure of intermediate sequences which obstruct hslp76’s approaching hItk to a certain extent (Supplemental data Fig. 2). Presenting a shape of Water Drop, the 3D structure of bbslp76 is also compacted and rigid (Fig. 7, the 3^rd^ column), in which the potential protein binding sites could not be exposed completely (Supplemental data Fig. 2). When we deleted the bbWW domain in bbslp76, ΔWW-bbslp76 presents an arc-shaped structure which is much more stretched than the Water Drop Shape (Fig. 7, the 4^th^ column) and the distribution of the binding motifs resembles that of hslp76 (Supplemental data Fig. 2). These results indicated that the bbWW domain made the 3D structure of slp76 rigid and closed, which may influence a web of protein interactions with bbslp76 and thereby the formation and function of the slp76 signalosome.

### 3D model comparison of slp76 from several keystone species

**Figure 8:**
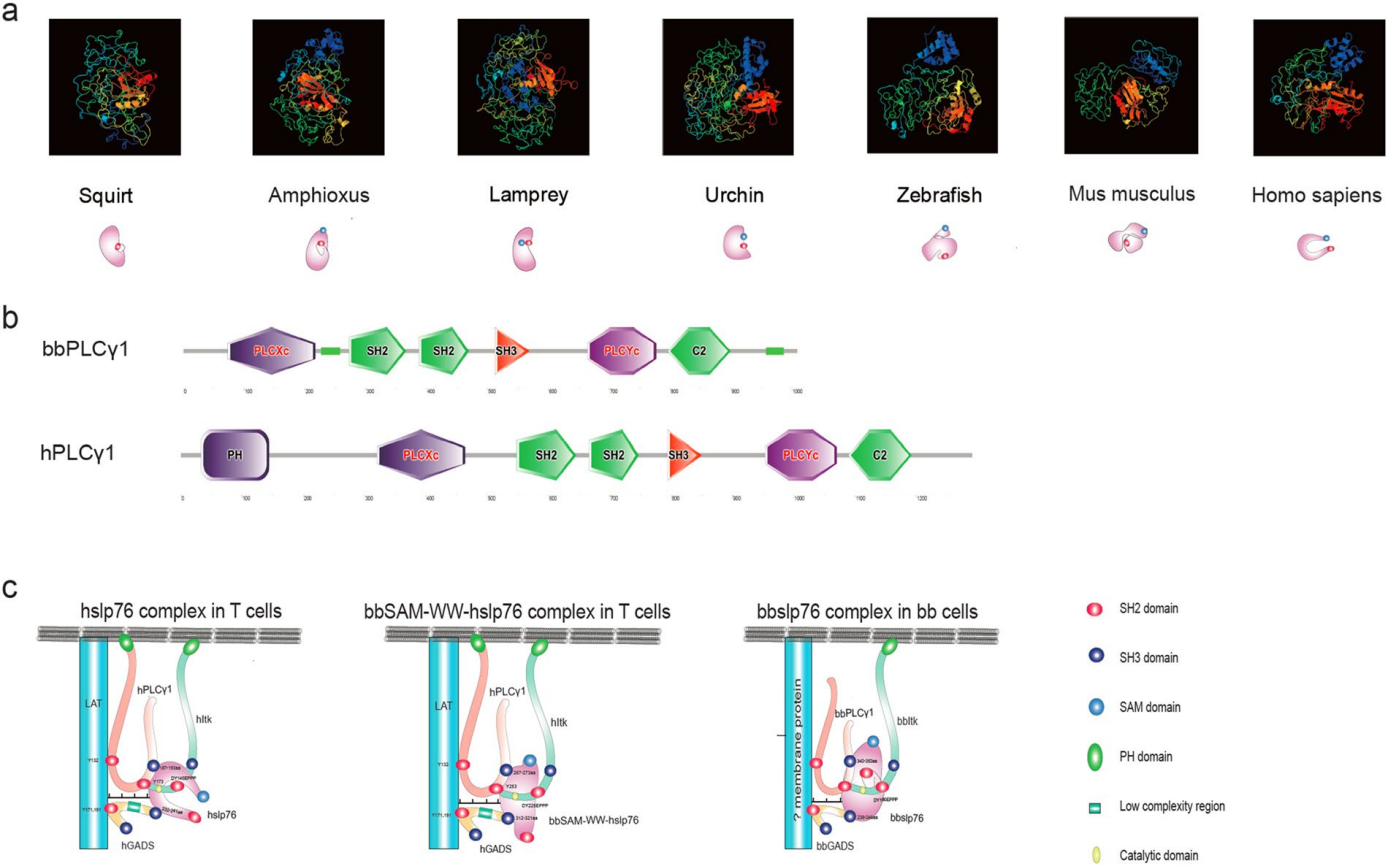
3D model comparison of slp76 from several keystone species. (**a**) 3D model comparison of slp76 from several keystone species. The 3D structures were predicted by Phyre2. (**b**) Protein structures of bbPLCγ1 and hPLCγ1generated by SMART. (**c**) A web of interactions connects hslp76 to the hPLCγ1, hItk, hLAT and hGADS. Predicted interactions of bbSAM-WW-hslp76 in T cells and bbslp76 in bb cells. Scales show the distance between LAT and slp76.

The 3D structure is very important for protein functions, especially for adaptor proteins which promote the formation of the protein signalosome acting as a connecting link between the preceding signaling transduction and the following signaling pathway. To understand the evolution of slp76 structure, we analyzed the sequences (Supplemental data Fig. 3) and 3D structures of slp76 from several keystone species (urchin, amphioxus, squirt, lamprey, zebrafish, Mus musculus and Homo sapiens), the results from which indicated that the 3D structure of slp76 evolved from strong contraction to stretch in accordance with the evolutionary status of keystone species except sea urchin (Fig. 8a). Although sea urchin is an ancestral organism, the genome of sea urchin is kin to human^34^, so it is reasonable that the 3D model of sea urchin slp76 is more similar to hslp76 (Fig. 8a).

In this study, our results suggested that slp76 signalosome proteins must have co-evolved to function properly. To further understand their co-evolution molecular mechanism, we analyzed the sequences and domains of bbItk and bbPLCγ1. The domain structure of bbItk is the same as hItk (Fig. 4d). Compared with hPLCγ1, bbPLCγ1 is shorter and has no PH domain that is responsible for the membrane localization of proteins^35^ (Fig. 8b), so we speculated that unknown membrane bound protein may recruit bbGADS, bbslp76 and bbPLCγ1 for their translocation to the cytomembrane in amphioxus cells. Next, based on the sequences and the reported interaction motifs of hslp76 signalosome proteins^19, 32, 36^, we also reconstructed an interaction schematic of hslp76, hLAT, hGADS, hPLCγ1 and hItk (Fig. 8c, left) and a schematic of the slp76 signalosome when hslp76 is replaced by bbSAM-WW-hslp76 (Fig. 8c, middle). In addition, based on the homology motifs analysis, we reconstructed a schematic of the bbslp76 signalosome in amphioxus cells (Fig. 8c, right). From these schematics, we can see that slp76 evolved to a more open and flexible structure, and thus could supply more space facilitating its interaction with more partners. In detail, in the hslp76 signalosome, the hslp76 structure facilitates the complex formation of hLAT, hGADS, hslp76, hPLCγ1, and hItk flexibly and neatly. The space between hLAT and hslp76 is large enough to recruit hPLCγ1 and membrane translocation (Fig. 8c, left). In the bbslp76 complex, bbGADS is shorter than hGADS due to the lack of a LCR (Fig. 3b), so the space between the unknown membrane protein and bbslp76 is smaller but suitable for the shorter bbPLCγ1 (Fig. 8c, right). In the bbSAM-WW-hslp76 complex, the location of the protein binding sites was changed so the complex may not be stable (Fig. 8c, middle). In conclusion, during evolution, the slp76 signalosome requires the co-evolution of its components and their proper assemble to adapt to the cellular functions in different species.

## Discussion

In this study, we explored the slp76 signalosome from an evolutionary perspective. Detecting the binding of bbslp76 with bbItk as well as bbGADS, we demonstrated that slp76 complex exists in amphioxus. Although the complex is conserved, the signalosome proteins have altered in size or structure. Notably, bbslp76 differs in its structure with a unique WW domain. Next, the functions of the bbslp76 complex were investigated in Jurkat TAg cells. Following TCR stimulation, compared with hslp76, bbslp76 enhanced total tyrosine phosphorylation level or NFAT transcriptional activity to a much lesser extent due to its entirely dampened interaction with hItk, and this deficiency could not be rescued by bbGADS. Moreover, bbGADS failed to associate with hslp76 or hLAT. Our mapping results revealed that the replacement of hSAM domain with the bbWW domain (bbWW-hslp76) mimicked bbslp76 in the impairment of hItk-association and NFAT activation, while the deletion of the WW domain in bbslp76 (ΔWW-bbslp76) reversed the inhibitory effect of bbslp76. Therefore, it is manifested that bbWW domain plays the biggest part in altering slp76’s conformation, partly contributing to a distinct interaction model within the complex in amphioxus. It is worth noting that squirt slp76 contains neither SAM nor WW domains, both of which have not emerged until bbslp76, to the best of our knowledge. It is reported that both SAM and WW domains can mediate protein-protein interactions^37, 38^, however, instead of the WW domain, the SAM domain has been reserved during the following evolution, indicating that WW did not fit well in the slp76 signalosome. We failed to find any GADS-like adaptors in squirts, implying their lack of slp76 signalosome archetype. According to the alignment of the predicted 3D structures of slp76 and the sequential analysis of GADS as well as PLCγ1 from several keystone species, we assume that slp76 signalosome molecules have co-evolved to work properly in accordance with functional needs.

Compared with hslp76, bbSAM-WW-hslp76 is more compact with two terminals sitting distal from each other, which may occupy the space where hItk intended to be and impair its accessibility to the signalosome. Similarly, the C-terminal of bbslp76 is surrounded by its intermediate motifs, which may hinder hItk and hGADS from approaching the binding sites. To further explore how slp76 complex evolved, we examined bbGADS’s association with hslp76 and hLAT with no obvious interactions being observed. Composed of a central SH2 domain flanked by two SH3 domains, GADS is a hematopoietic-specific member of growth factor receptor bound-protein 2 (GRB2) adaptor family. According to the predicted 3D structure, bbGADS seems too short to interact with hslp76 and hLAT as hLAT-bound hPLCγ1 is larger than bbPLCγ1. bbPLCγ1 does not contain the PH domain responsible for its membrane binding like hslp76, suggesting that it became easier for PLCγ1 to be activated in evolution. It is reported that LCR is involved in forming novel genes, protein structures and functions^39–41^. Our sequence alignments showed that hslp76 lost some LCRs yet hGADS gained a LCR, implying LCRs can regulate adaptors and signalosome formation through its gain and loss. We speculate that to meet the needs of cellular function evolution and better serve a complex LCR adjusts itself in a flexible pattern within the adaptors.

Our results demonstrated that the slp76 signaling complex exists as early as in chordate invertebrate lineage. In amphioxus, bbslp76 binds to both bbItk and bbGADS. However, we did not find any LAT like molecules in amphioxus. There may be several possibilities: one is that there might be an unknown membrane-bound molecule having the same function as LAT to recruit slp76 signalosome in amphioxus. It is reported that in the absence of LAT, slp76 can function and translocate to the immunological synapse^42^, that is to say, there are alternative ways to activate hslp76. This alternative route for slp76 activation may exist in amphioxus and be more conserved than the LAT-nucleated pathway. Thus, seeking the upstream regulator or recruiter of bbslp76 in amphioxus can facilitate us the better understanding of the slp76 signalosome in humans. Another possibility is that there is no LAT or other molecules recruiting slp76 in amphioxus, and the bbslp76 complex is just awaiting the presence of LAT in the following course of evolution.

In mammals, the slp76 signalosome exhibits TCR-induced formation. As we know, the antigen-responding signaling cascades in an immune response is crucial to maintaining homeostasis and preventing overreaction in resting T cells as well as saving energy. Interestingly, we observed the association of bbItk-bbslp76 or bbItk-hslp76 or bbslp76-hPLCγ1 in resting T cells, however the binding was irresponsive to TCR stimulation, unlike TCR-stimulation enhanced associations among hslp76, hItk and hPLCγ1. This finding suggests that highly developed organisms evolved finer and securer signaling regulation in part by better structural regulation performance and post-translational modifications of signal molecules. Intriguingly, WW deletion recovered the stimulation-responding association between bbslp76 and hPLCγ1, which may result from the rescue of bbslp76’s binding to hItk. Given the fact that Itk is the core enzyme for PLCγ1 activation and slp76-Itk association is required for Itk kinase activity, this potential Itk-dependent amplified interaction between slp76 and PLCγ1 after TCR ligation presumably serves as a threshold of PLCγ1 activation.

In conclusion, we hypothesize a model for the evolution of the slp76 complex. During the time course from invertebrate lineage to vertebrate lineage, slp76 lost its WW domain and some LCRs to pack itself into a more stretched conformation. Meanwhile, GADS gained a LCR evolving into a bigger scaffold. These variations occurred to meet the requirement of a larger space between LAT and slp76 for recruiting effector kinases with better efficiency and accuracy.

## Materials and Methods

### Plasmids and Reagents

hslp76 (NCBI Reference Sequence: NP_005556.1), hPLCγ1 (NCBI Reference Sequence: NP_002651.2), hGADS (NCBI Reference Sequence: NP_001306364.1), hItk (NCBI Reference Sequence: NP_00537.3) and hLAT (NCBI Reference Sequence: NP_001014989.2) cDNAs were amplified by PCR from a cDNA library of Jurkat E6.1 cells. hslp76 cDNA was cloned into pEF6Myc/his B (Invitrogen). The amphioxus slp76-like sequence reported in this article has been submitted to GenBank (http://www.ncbi.nlm.nih.gov/genbank/) under accession number KX430178. hPLCγ1, hGADS, hItk and hLAT. cDNAs were cloned into pcDNA3.1-HA/GFP (Invitrogen), respectively. *B. Belcheri* bbslp76, bbItk, bbGADS, bbPLCγ1 were identified using hslp76, hItk, hGADS and hPLCγ1 as BLAST queries against the database of *B. Belcheri* genome (http://mosas.sysu.edu.cn/genome) respectively. bbslp76, bbGADS and bbItk were cloned from Chinese amphioxus cDNA by a specific primer pair derived from identified sequences of bbslp76, bbGADS and bbItk. Fulllength bbslp76, bbslp76 mutants with the deletion of the WW domain (ΔWW), and chimeric proteins bbSAM-hslp76, bbWW-hslp76, bbSAM-WW-hslp76, and bbSH2-hslp76 cDNAs were then subcloned into pEF6Myc/his B (Invitrogen). bbGADS and bbItk cDNAs were subcloned into pcDNA3.1-HA/GFP (Invitrogen). To construct shRNA vectors, 60-bp hairpin oligonucleotides were designed and subcloned into pSUPER.retro.neo vectors (OligoEngine). hslp76 was targeted by the following sequences: 5′-GGTCAATGGACAAACAC-3′ in hslp76 mRNA UTR region and 5′-UUCUCCGAACGUGUCACGU-3′ was used as a nonspecific negative control. All constructs were confirmed by DNA sequencing. The rabbit antibody to HA (sc-805) and the mouse antibodies to Myc (sc-40), β-actin (sc-47778), GFP (sc-9996), phosphorylated Erk (Thr202/Tyr204) (sc-16982) and SLP76 (sc-13151), as well as HRP-conjugated secondary antibodies were purchased from Santa Cruz Biotechnology (Santa Cruz, CA, USA). Phosphotyrosine specific mAb (pY100) was purchased from Cell Signaling Technology (Danvers, MA, USA). Mouse antibodies to human α-CD3 and α-CD28 were from eBioscience. DAPI, Alexa Fluor 488- and 594-labelled secondary antibodies were from Molecular Probes and poly-L-lysine was from Sigma.

### Protein purification and preparation of polyclonal antibody

Recombinant bbslp76 (1-318aa) fused with a His tag was constructed into pET-28a+ and expressed as a soluble protein in BL21 (DE3). Briefly, bacteria were grown at 37 °C in LB with 50 μg/ml kanamycin to 0.8 OD (optical density at 600 nm) and induced with 1 mM isopropyl-β-D-thiogalactoside at 18 °C for 20 h. Bacteria were collected and resuspended in His-binding buffer (20 mM Na_3_PO_4_, 0.5 M NaCl, 20 mM imidazole, pH7.4), and after ultrasonic breakage for 15 min, the supernatant was transferred to the Ni sepharose-preloaded column (Promega) with a linear flow rate of 50 cm/h. Then, the soluble protein was washed with His-binding buffer until the absorbance reached baseline, and was finally eluted with His-elution buffer (20 mM Na_3_PO_4_, 0.5 M NaCl, 250 mM imidazole, pH7.4) using a step gradient. The concentration of purified protein was determined according to standard protein BSA. For polyclonal antibody preparation, 100–200 μg of purified bbslp76 emulsified with Freund’s complete adjuvant (Sigma) was injected intraperitoneally into a BALB/C mouse and after two weeks 50–100 μg of purified bbslp76 emulsified with Freund’s incomplete adjuvant (Sigma) was injected intraperitoneally into a BALB/C mouse once a week for three weeks, finally, approximately 200 μl of sera was collected from a mice orbital sinus.

### Immunostaining for microscopy and quantitative real-time PCR

For microscopy, amphioxus intestine or gill cells plated on poly-L-lysine coated coverslips were fixed with PBS plus 4% paraformaldehyde at room temperature for 15 min, then permeabilized with PBS plus 0.2% Triton X-100 for 4 min. Nonspecific binding was blocked by incubating with PBS plus 2% BSA for 30 min, and amphioxus intestine or gill cells were stained with anti-bbslp76 polyclonal antibodies at 4 °C overnight. The coverslips were rinsed with PBS three times for 10 min followed by incubation with Alexa 488-coupled chicken anti-mouse (A-21201, Invitrogen) at room temperature for 1 h. Rinsed with PBS three times for 10 min, cells were then stained with 0.2 μg/ml DAPI for 10 min in the dark, mounted in Mowiol (Calbiochem), and further analyzed by confocal microscopy (Leica TCS-SP5) with a 63× (N.A. 1.3) glycerol-immersion objective lens (Leica)^43^. Quantitative real-time PCR was performed in a total volume of 10 μl with 5 μl of 2× realstar green power mixture (Genstar), 0.2 μl of primers, 0.5 μl of cDNA templates and double-distilled water. Real-time PCR was performed in LightCycler® 480 System (Roche). Data were collected and quantified using the 2-ΔΔCt method according to the Ct values of target genes and normalized to endogenous control 18S RNA. The results were shown as the mean ± SEM with three replicates analyzed using a two-tailed t test with significance for P < 0.05.

### Cell Culture and Transfection

The human leukemia Jurkat TAg cell line of simian virus 40 large T antigen transfected Jurkat TAg cells was grown in RPMI1640 medium (Invitrogen) supplemented with 10% fetal bovine serum (FBS, Hyclone, Logan, UT, USA), 100 U/ml streptomycin, and 100 U/ml penicillin (Gibco) at 37 °C, 5% CO2. Jurkat TAg cells (4 × 10^6^) were washed twice, resuspended in serum-free RPMI 1640 medium, and transiently transfected with a total of 5 μg of DNA or plus 200 nmol siRNA or plus 5 μg of pSUPER-NC or pSUPER-shslp76 by Amaxa nucleofector device (Lonza, Allendale, NJ, USA). Conditions for human CD4+ T cells transfection were recommended by the manufacturer. After transfection with the siRNA mixture, cells were incubated in RPMI medium containing 10% FBS without penicillin or streptomycin. At 24 hours or 48 hours (siRNA knockdown experiments) later, transfected cells were used in the experiments. siRNA oligonucleotides were purchased from GENEPHARM (Suzhou, China). The sislp76 sense-strand sequence is as follows: 5′-GGTCAATGGACAAACAC-3′. DH5α and BL21 (DE3) were grown in Lysogeny broth (LB) supplemented with ampicillin at 100 μg/ml or kanamycin at 50 μg/ml, accordingly.

### Activation, Immunoprecipitation and Immunoblotting

Cells were washed with serum-free RPMI1640 medium, serum starved, incubated on ice for 20 min, then stimulated with antihuman CD3 (10 μg/ml) at 37 °C for the indicated times with gentle shaking by crosslinking with a secondary donkey anti-mouse IgG (10 μg/ml, Santa). After washing in ice-cold PBS, cells were lysed in lysis buffer (20 mM Tris-HCl, pH 7.5, 150 mM NaCl, 5 mM EDTA, pH 8.0, 5 mM NaPPi, 1 mM sodium orthovanadate (Na_3_VO_4_), 1 mM PMSF, 1% NP-40, and 10 μg/ml each aprotinin and leupeptin). Lysates were incubated with the indicated antibodies plus 30 μl of protein G PLUS-agarose (GE healthcare) overnight at 4 °C with gentle shaking. Samples were washed five times with lysis buffer, and the immunoprecipitates (IP) were dissolved in 2X Laemmli buffer, subjected to SDS-PAGE, transferred to PVDF membranes, and immunoblotted with the indicated antibodies^44^. Densitometry of the bands was quantitated by Quantity one software.

### Luciferase Reporter Assays

For reporter assays, Jurkat cells were transfected in triplicate by electroporation with a combination of NFAT luciferase reporter plasmid and the indicated plasmids. After 48 hours, cells were either unstimulated or stimulated with crosslinked anti-CD3 plus-CD28 mAbs (1 μg/ml, respectively) for 6 hours, lysed, and collected for luciferase reporter assay on Berthold Lumat LB 9507. The luciferase activity of cell lysates was measured with a Promega luciferase assay kit according to the manufacturer’s instructions. Co-transfected Renilla luciferase reporter plasmid was used as an internal control.

## Electronic supplementary material

Supplementary figures
