# Supplementary material for: Molecular mechanisms underlying the evolution of the slp76 signalosome
Source: Sci Rep. 2017 May 4;7:1509. doi: 10.1038/s41598-017-01660-0 (PMC5431462; doi:10.1038/s41598-017-01660-0)
Supplement: Supplementary file 1 — Supplementary figures [file 41598_2017_1660_MOESM1_ESM.doc]

**Molecular mechanisms underlying the evolution of the slp76 signalosome**

**Xuemei Qu^1, #^, Xin Lan^1, #^, Chong Deng^1^, Jiatao Zhou^1^, Jingjing Du^1^, Shengfeng Huang^1^, and Yingqiu Li^1*^**

^1^State Key Laboratory of Biocontrol, Key Laboratory of Gene Engineering of the Ministry of Education, College of Life Sciences, Sun Yat-Sen University, Guangzhou 510275, People’s Republic of China;

^#^XM.Q. and ^#^X.L. contributed equally to this work.

*Address correspondence and reprint requests to Dr. Yingqiu. Li., College of Life Sciences, Sun Yat-Sen University, Guangzhou 510275, People’s Republic of China. E-mail address: lsslyq@mail.sysu.edu.cn

Supplemental data Figure1. bbslp76 interacts with bbGADS and bbItk respectively. (a) bbslp76 interacts with bbGADS in T cells. Jurkat TAg cells were transfected with the indicated constructs. After 48 hrs, cells were stimulated with or without CD3 (10 μg/mL); then, lysed and lysates were split into two parts, one part of the lysates was subjected to a Myc IP (immunoprecipitation), and the other part was used for WCL (whole cell lysis, 40 μL). Samples were studied by western blot, and probed with indicated antibodies. (b) The interaction of bbslp76 with bbItk in T cells. Cells and lysates were prepared as in (a). Samples were studied by western blot, and probed with indicated antibodies.

Supplemental data Figure2. Key protein binding motifs of hslp76, bbSAM-WW-hslp76, bbslp76 and ΔWW-bbslp76.

Supplemental data Figure 3. The structures of slp76 from several keystone species generated by SMART.
